# Multiple host-plant use may arise from gender-specific fitness effects

**DOI:** 10.1673/1536-2442(2006)6[1:MHUMAF]2.0.CO;2

**Published:** 2006-04-21

**Authors:** Melanie Gibbs, Lesley A. Lace, Martin J. Jones, Allen J. Moore

**Affiliations:** 1 Department of Biological Sciences, Manchester Metropolitan University, U.K; 2 School of Biological Sciences, University of Manchester, U.K

**Keywords:** Host species, *Pararge aegeria*, larval development

## Abstract

Ovipositing females are predicted to select host-plants that will maximise offspring survival and fitness. Yet hosts often differ in the component of larval fitness affected so host-selection often involves a trade-off between short development times and large size and high fecundity of offspring. If host-species can directly affect development rates and body size, and if there are gender differences in resource allocation during development, there can be different sex-specific selection pressures associated with different hosts. Using a Madeiran population of the speckled wood butterfly Pararge aegeria (L.) as the model species gender differences in larval development and size were examined in response to the hosts Brachypodium sylvaticum, Holcus lanatus and Poa annua. It was observed that male and female P. aegeria larvae differed, with their responses dependent on the host species. These results would suggest that oviposition behavior is a complex process, and use of multiple hosts may have evolved to balance the conflicting needs of male and female larvae. Co-evolution of host selection and oviposition behaviors may help to balance the differing performance needs of offspring.

## Introduction

Oviposition behavior of females is hypothesised to have evolved to ensure that the host-plant species selected will maximise larval growth and survival (Thompson & Pellmyr 1991). Ovipositing females are thought to show a preference for host-plants that support rapid larval development (Janz et al. 1994; Nylin & Gotthard 1998; Wedell et al. 1997), as short development times act to reduce exposure times to predators and parasitoids (Connor 1991; Klok & Chown 1999), and facilitate rapid reproduction in growing populations (Fischer & Fiedler 2000; Gotthard et al. 1994). However, hosts can also affect offspring body size (Janz et al. 1994; Nylin & Gotthard 1998; Roff 1992; Steams 1992), therefore, host-plant selection at oviposition may involve a trade-off between plants that support rapid offspring development, and plants that enable offspring to achieve a large size/high fecundity.

Further, oviposition site choice is complex because there are many factors that may impact on larval fitness. Factors that may influence the host-plant choice of females are thought to include the density, diversity and distribution of vegetation surrounding host-plants (Papaj & Rausher 1983), the amount of nutrition the host will afford for larval growth (Leather & Burnand 1987; Ostaff & Quiring 2000), host damage (Wise et al. 2002), host-plant-size (Kirk 1991), leaf texture (Lance 1983), the plant part selected (Jallow & Zalucki 2003), host-plant age (Hunter & Elkinton 1999; Lamb et al. 2003; Smyth et al. 2003) and host abundance (Janz et al. 1994; Nylin & Janz 1996). Additionally, host-plant selection by individual females can vary geographically (Lance 1983) and temporally (Tabashnik et al. 1981), and thus offspring survival is also spatially and temporally variable (Van Nouhuys et al. 2003).

Studies with phytophagus insects reveal that there is a large variation in the performance of individuals on different hosts (Lance 1983; Singer et al. 1991; Tabashnik et al. 1981; Tikkanen et al. 2000; Wedell et al. 1997). Wedell *et al.* 1997 observed that the larval host-plant of the comma butterfly ( Polygonia c-album) strongly affected the propensity to enter diapause, and that males tended to enter diapause to a greater extent than females when reared on poor host-plants. Tikkanen *et al.* 2000 found that Operophtera brumata females had higher growth rates than males, and that the host-plant species affected the weight of adult females, but not the weight of adult males.

Given that host-plant species can directly affect development rates or body size, male and female larvae may face different life history trade-offs on different hosts. A sexual dimorphism in size occurs in the speckled wood butterfly, Pararge aegeria, due to differences in the allocation of larval derived resources (Sibly et al. 1997). Sibly *et al.* 1997 suggested that males may invest in lipid reserves to enable longer mating flights or territorial disputes (Sibly et al. 1997), whereas females invest in nitrogen reserves, which are allocated to the abdomen for reproduction (Karlsson & Wickman 1990). Since P. aegeria feed on a more protein-rich food source during their larval stage than during their adult stage (Svärd & Wiklund 1989) and because spermatophores contain only a small percentage of protein, therefore providing unsubstantial nuptial gifts (Boggs 1981), there is little opportunity for adult females to accumulate additional nitrogenous resources for reproduction. Males, however, can gain additional lipid resources through adult feeding. Thus, female fecundity is largely dependent on the resources accumulated during the larval stage (e.g. Wiklund et al. 2001). Differences in host-plant utilisation during larval development, perhaps due to nutritional differences between host species, may therefore carry a higher cost for female P. aegeria than for male P. aegeria, as large body size is expected to be more important for adult females than adult males.

Given the propensity of P. aegeria to use more than one host, and the gender differences in larval resource allocation, the hypothesis was tested that male and female larvae differ in their relative performance on different hosts. Larvae were reared on B. sylvaticum, H. lanatus and P. annua and the effects of host plant species on development time, pupal mass and survival in a Madeiran population of P. aegeria were examined. Pararge aegeria is an important model system for insect life history and ecology and Northern European populations of P. aegeria have been extensively studied (e.g. Gotthard et al. 1994; Van Dyck et al. 1997; Gotthard et al. 2000; Van Dyck & Wiklund 2002). Although the natural history of host plant use and larval densities has not been extensively studied in natural populations of P. aegeria on Madeira, it is known that although the preferred host plant B. sylvaticum is very widespread and abundant, eggs laid are not always uniformly distributed, with large numbers of eggs sometimes observed on single host plants (Jones et al. 1998). By examining the effects of host plant species on gender specific life-history and fitness in another population of P. aegeria from a very different environment, this study aims to add additional information to the extensive knowledge of the life history of this species.

## Materials and Methods

### Study organism

In 1999 at Portela on Madeira, 50 eggs of P. aegeria were collected from Brachypodium sylvaticum, and returned to the Manchester Metropolitan University butterfly house for rearing. 36 adults eclosed and were maintained for three generations in a flight cage 1.25 × 3.90 × 1.80m. Adults were fed daily with a 10% honey solution via five artificial flowers (for design see Cory & Goulson 1993), distributed at random in the flight cage. Honey supplies were replenished daily. Ten Brachypodium sylvaticum plants were made available for the F3 generation females (n = 24) to lay their eggs. These plants were distributed randomly throughout the flight cage. Photoperiod (Nylin et al. 1995) and temperature (Sibly et al. 1997) are known to affect the development of P. aegeria larvae. A 12:12 h LD cycle, a temperature of 21 ± 2° C and a humidity of 50 ± 10% were therefore strictly maintained for the whole of the growth period (i.e. from egg stage to adult stage). Lighting in the butterfly house was provided at an intensity of 1500 Lux by eight ceiling lamps.

### Host-plants

The larval host-plants B. sylvaticum, H. lanatus and P. annua were grown from commercially produced seed, and sown in 4-inch pots containing soil-based compost. To reduce the affect of environmental variation on host plant growth, all of the plants were reared under identical conditions at a temperature of 23 ± 2° C and a humidity of 45 ± 10%. Light was provided at an intensity of 7000 Lux over a 16:8h LD cycle. Each of the plant species responded well to these standardised growth conditions. Plants were watered daily, but never fertilised. Host-plant age is known to affect the quality of the plant as a resource for larvae, (Scriber & Slansky 1981), and can act to lengthen larval development (Hunter & Elkinton 1999). Therefore, all of the plants used in this study were of young ages (i.e. were used when they had between 50 and 70 blades).

### Experimental design

Eggs were collected daily from F3 generation females maintained in the laboratory population. These eggs were removed from the host-plants and single eggs were placed into individual 8 ml transparent containers until hatching. Upon hatching, the larvae were randomly assigned to potted plants of either B. sylvaticum, H. lanatus or P. annua at densities of 10 larvae per plant. A total of 150 larvae were distributed at this density over 15 plants. Older larvae have been shown to have a significant competitive advantage (Briggs et al. 2000; Krebs & Barker 1995), therefore all of the larvae assigned to an individual plant shared the same hatching date, although not the same parent, thus families were randomly assigned across treatment groups. Plants were similar in size with approximately 50 blades per plant. The individual plants were placed into separate 0.5 m^3^ netted cages in the butterfly house. To avoid food shortages, all plants were changed before they had been completely defoliated.

Pupation date, pupal mass and eclosion date were recorded for each individual. At pupation, individuals were removed from their host-plant and suspended (by a cotton thread) in separate 38 ml transparent plastic containers until eclosion. Upon emergence, the sex of the adult was recorded.

### Statistical analyses

A two-way analysis of variance, with replicate plants nested under species, was used to investigate the effects of host-species, gender and the host species x gender interaction on larval development time, pupal time, pupal mass and larval growth rate. Nesting replicate plants under a given species controlled for measures from more than one individual derived for a given plant (a plant effect). Larval growth rate (mg/day) was calculated by dividing pupal mass (mg) by larval development time (days). A Pearson chi-square test was used to compare the proportion of surviving adults on each host-plant, and to determine whether there were host-plant dependent differences in adult sex-ratio. A logistic regression was performed to determine how the size of the individual at pupation affected survivorship to adulthood. All tests were two-tailed. The statistical procedures were performed using Systat 9.0.

## Results

### Development time

There was an overall significant effect of host-plant species on the length of the larval growth period, but no effect of gender and no gender x host-plant interaction. Larval development time was shortest when offspring were reared on H. lanatus and longest when reared on P. annua (Table 1).

**Table 1.**
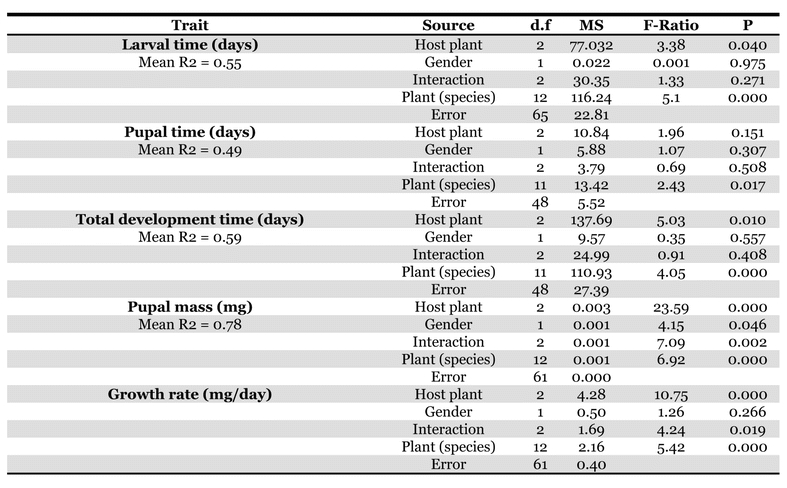
Nested Two-way ANOVA for effects of host-plant and gender on life history traits in Pararge aegeri

There was no effect of host-plant on the amount of time spent as pupae, and no effect of gender (Table 1).

Total development time (larval time + pupal time) was significantly affected by host-plant species, but there was no effect of gender and no gender x host-plant interaction. Total development time was observed to be shortest when larvae were reared on H. lanatus, and longest when larvae were reared on P. annua (Table 1).

### Pupal mass

Pupal mass was significantly affected by host-plant species, gender, and there was also a significant gender x host-plant interaction (Table 1). Both sexes showed a general trend of small pupal mass on B. sylvaticum and large pupal mass on P. annua, but the response to H. lanatus was different between the sexes (Table 2). Females attained larger pupal mass than males when reared on H. lanatus.

**Table 2.**
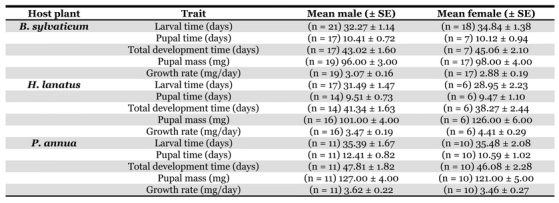
Life history data for male and female P. aegeria larvae reared on different host plants.

### Growth rate

**Figure 1.**
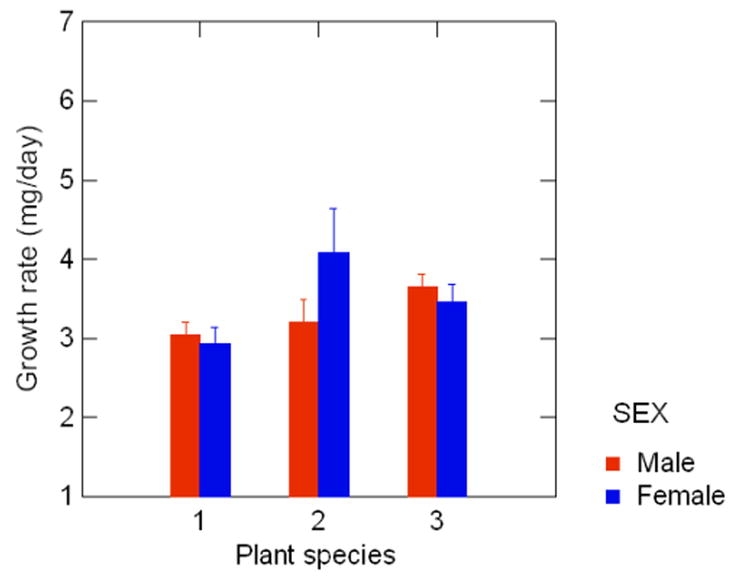
The effects of host-plant and gender on growth rates (mg/day) in male and female P. aegeria larvae where plant species 1 = 5. sylvaticum, 2 = H. lanatus and 3 = P. annua

Growth rate was significantly affected by host plant species, and there was a significant gender x host-plant interaction, but no affect of gender alone (Table 1). Males had faster growth rates than females on B. sylvaticum and P. annua, but on H. lanatus females had faster growth rates than males (Figure 1).

### Survivorship of adults

There was no effect of host-plant on survival to pupation (Table 3; χ^2^ = 3.470, d.f. = 2, P = 0.176) or eclosion (Table 3; χ^2^ = 4.376, d.f. = 2, P = 0.112). There was however, a significant relationship between size at pupation and likelihood of surviving to adulthood (logistic regression; χ^2^ = 12.674, d.f. = 1, P = < 0.001).

## Discussion

Life-history theory predicts that an optimal host-plant will facilitate short growth times and large size, and also maximise survival rates. Life history studies also show a trade-off between size and rate of development (Janz et al. 1994; Nylin & Gotthard 1998; Roff 1992; Steams 1992). In this study, a gender difference was found in response to different host plant species. Males had the highest growth rates when reared on P. annua, whereas females had the highest growth rates when reared on H. lanatus. Given the unequal fitness effects of host-plants on male and female larvae, ovipositing P. aegeria could use a mixture of host-plants, as a ‘bet-hedging’ strategy.

On Madeira, the preferred host plant B. sylvaticum is widespread and abundant (Owen et al. 1986, Jones et al. 1998). In the laboratory, under crowded conditions, development on B. sylvaticum potentially carries a fitness cost for female larvae because females do not achieve high growth rates on this species compared to H. lanatus and P. annua. It is known that under optimal conditions female P. aegeria have higher growth rates than males (Gibbs et al. 2004) and selection for large female size (and hence high fecundity) would appear to be more important for fitness than selection for large male size (Gotthard et al. 1994; Leimar et al. 1994). Given the strong selection for large size in females, this might suggest a possible parent-offspring conflict between oviposition preference and female offspring performance. However, further studies designed to examine this specific idea are required, especially given that, in nature, an oviposition preference for B. sylvaticum may enhance larval fitness through other factors (e.g. density of vegetation surrounding host plants, Papaj & Rausher 1983) that are controlled for during laboratory studies. Also, during this laboratory experiment offspring performance was examined under sub-optimal conditions (larval crowding) where larvae experienced periods of food shortage. High growth rates have been predicted to be costly in terms of fitness (e.g. increased mortality) during periods of food shortage (Gotthard et al. 1994). Offspring performance, particularly female performance, is therefore likely to be enhanced under more optimal conditions, where the costs associated with a high growth rate would be reduced. For example, it is known that when reared under solitary conditions on B. sylvaticum, female offspring have higher growth rates than when reared in groups, and they also have higher growth rates than males (Gibbs et al. 2004). Studies examining the costs associated with high growth rates deserve further attention, particularly in relation to how oviposition behavior could act to minimise the effect. For example, if ovipositing females avoided crowded egg-laying sites, and laid single eggs on host plants, they may act to; 1) buffer the size disadvantage incurred by their female offspring starting life on a poor host-plant 2) increase larval survival to adulthood 3) decrease the magnitude of the female preference-performance conflict and 4) reduce male-female larval competition. To achieve this, ovipositing females would need to devote time to searching for optimal host plants and locations.

Plant phenology may have a strong affect on offspring performance, and it would be interesting to examine plastic responses to host species of P. aegeria within natural populations. More specifically, further studies are recommended to explore host plant niches with respect to environmental conditions, host plant use during oviposition, and larval densities typically observed on different host species on Madeira. Such studies may provide a valuable insight into the evolution of life history trade-offs in P. aegeria.

This study has shown that there are gender-related differences in larval performance on different hosts, and that host-plant selection may carry fitness consequences to offspring. However, the host-plant species selected for oviposition is not the only factor that may influence larval fitness, and adult females may be able to buffer any disadvantages through other behaviors during oviposition (e.g. by avoiding egg clumping). The co-evolution of host-specificity and oviposition behaviors may have helped to balance the conflicting needs of female offspring, male offspring and their mother.
